# The recent advancements of single-cell RNA sequencing in pre-eclampsia

**DOI:** 10.3389/fmed.2026.1774379

**Published:** 2026-04-10

**Authors:** Shenglan Tang, Xiao Lin

**Affiliations:** 1 Department of Obstetrics and Gynaecology, Affiliated the Third People’s Hospital of Suining City, Suining, Sichuan, China; 2 Department of Obstetrics, Affiliated Hangzhou First People’s Hospital, School of Medicine, Westlake University, Hangzhou, Zhejiang, China

**Keywords:** mechanisms, pre-eclampsia, pregnancy, single-cell RNA sequencing, treatment

## Abstract

Preeclampsia (PE) is a multisystem syndrome that manifests after 20 weeks of gestation, with a global incidence of 2%–8%, and is one of the leading causes of maternal and perinatal mortality. Its etiology is complex, involving multiple mechanisms such as abnormal placentation, immune dysregulation, and angiogenic imbalance, with early-onset PE (EOPE) and late-onset PE (LOPE) exhibiting distinct pathological foundations. In recent years, the application of single-cell RNA sequencing (scRNA-seq) has provided a novel perspective for deciphering the cellular heterogeneity and molecular mechanisms of PE. This review systematically summarizes the latest advances in scRNA-seq applications in PE research, focusing on how this technology reveals: (1) Dysfunction of trophoblast subpopulations and its association with defective spiral artery remodeling; (2) Dynamic changes in the immune microenvironment, including macrophage polarization, functional subsets of uNK cells, and T cell regulatory networks; (3) Cell-specific dysregulation of key signaling pathways; (4) The distinct cytopathological features of early-onset versus late-onset PE. Furthermore, scRNA-seq has facilitated the discovery of multi-gene-based early diagnostic models and potential therapeutic targets. Compared to traditional bulk sequencing, scRNA-seq enables the resolution of cellular heterogeneity, identification of rare cell subpopulations, and elucidation of intercellular communication networks. However, its limitations include difficulties in sample acquisition, high technical costs, complex data analysis, and challenges in capturing multinucleated syncytial structures. In the future, scRNA-seq is expected to provide highly promising therapeutic strategies for PE patients.

## 1 Introduction

Pre-eclampsia (PE) is a pregnancy-related disorder manifesting after 20 weeks of gestation, with severe ramifications for both the mother and fetus (1). The manifestations of this condition can be categorized into several distinct classifications, including maternal hypertension, proteinuria, and damage to the mother’s heart, kidneys, liver, or brain (2, 3). Other manifestations include uteroplacental dysfunction and intrauterine growth restriction (IUGR) (4). With a global incidence of 2%–8% (5), it poses a significant challenge to obstetricians. The classification of PE is based on the timing of its onset. PE is categorized into two distinct classifications, namely early-onset pre-eclampsia (EOPE) and late-onset pre-eclampsia (LOPE) (6). The former is defined as the onset of the condition before 34 weeks of gestation, while the latter is characterized by the onset of the condition after 34 weeks of gestation. Research indicates that various genetic, angiogenic, structural, and metabolic pathways are implicated in preeclampsia, including spiral artery remodeling, placental oxygenation, redox and immune tolerance at the maternal-fetal interface, and the balance between angiogenic and antiangiogenic factors. The available evidence suggests that EOPE arises from abnormal placental formation, secondary to defective spiral artery remodeling in the uterus (7, 8), the etiology of LOPE is thought to stem from an imbalance between placental aging and maternal susceptibility to cardiovascular and metabolic diseases (9–11).

Single-cell sequencing technology is a technique that utilizes molecular biology to sequence genetic information at the single-cell level. This process involves a series of steps, including single-cell isolation, lysis, amplification, and sequencing (12). In accordance with the objectives of the sequencing process, the following categorizations can be proposed: genome sequencing, transcriptomics sequencing, epigenomics sequencing, proteomics sequencing, and multi-omics integrated analysis (13, 14). Conventional sequencing is conducted at the multicellular level, yielding genetic data that represents the mean of multiple or diverse cell types (14, 15). Single-cell RNA sequencing (scRNA-seq) is a technique that addresses cellular heterogeneity and diversity to elucidate complex biological systems. This methodology facilitates the identification of rare cell types and the detection of molecular dysregulation associated with perturbations or diseases within individual cells. This finding unveils cell-specific regulatory mechanisms, thereby providing novel insights into normal tissue physiology, organ differentiation, and the pathophysiology of various disease processes (16–19).

In recent years, scRNA-seq has emerged as a valuable tool for studying the placenta in preeclampsia, offering a comprehensive approach to address cellular heterogeneity at the maternal-fetal interface. This paper summarizes the application of scRNA-seq in preeclampsia, offering novel insights for its diagnosis and treatment (20–22).

## 2 Preeclampsia

Hypertensive disorders are prevalent complications of pregnancy, placing women and their fetuses at disproportionate risk of further complications and lifelong sequelae. The severity of gestational hypertensive disorders varies, encompassing chronic hypertension during pregnancy, PE and chronic hypertension complicated by PE (23). Among these, PE carries the highest incidence and mortality rates, contributing to over 70,000 maternal deaths and 500,000 fetal deaths globally each year (24–26). Symptoms and signs are known to resolve postpartum in most patients, though some may experience persistent or progressive manifestations following delivery (23, 27). New-onset or persistent postpartum PE has emerged as a significant risk factor for perinatal morbidity (28) and is recognized as being associated with substantial cardiovascular disease (CVD) and cerebrovascular disease risk (29).

The etiology and pathogenesis of PE remain incompletely understood. PE is a multifactorial disorder involving multiple mechanisms and pathways, and cannot be explained by a single-factor theory. The predominant theory in this field is the “two-stage” model. Phase 1 is the preclinical stage, characterized by impaired trophoblast remodeling of the spiral arteries, leading to placental ischemia and hypoxia (30, 31). The establishment of uteroplacental circulation is typically completed by 12 weeks of gestation, while the invasion of intravascular and interstitial trophoblast cells is completed between 20 and 22 weeks of gestation, suggesting that this phase occurs between 12 and 22 weeks of gestation (32, 33). Phase 2 involves the secretion of soluble factors such as soluble vascular endothelial growth factor receptor 1 (sVEGFR-1) and soluble endoglin (sEng) by syncytiotrophoblasts, which trigger a systemic maternal inflammatory response, resulting in more extensive and severe clinical manifestations (30, 34).

Trophoblast cells migrate to the spiral arteries of the maternal uterus, forming vascular sinuses at the fetus-maternal interface to supply nutrients to the fetus. In the context of a normal pregnancy, this invasive process extends into the spiral arteries within the uterine myometrium, remodeling maternal arterioles into vessels characterized by high capacity and high flow (7). In placentas that are at risk of PE, trophoblast cells are unable to transition from the proliferative epithelial subtype to the invasive endothelial subtype. This results in incomplete spiral artery remodeling, maternal vascular stenosis, and subsequent relative placental (35).

In addition to uteroplacental insufficiency, inadequate uterine decidualization has also been demonstrated to influence the development of PE (1, 36–39). A plethora of studies have revealed the upregulation of hypoxia-inducible transcription factors (TFs) and hypoxia-related gene expression profiles in the placenta, alongside elevated expression of hypoxia-inducible factor (HIF)-1α and -2α in proliferative trophoblast cells and placentas from women with PE. This finding suggests that hypoxia may be a central factor in the pathogenesis of PE (40). Intermittent hypoxia and reoxygenation resulting from inadequate spiral artery invasion have been demonstrated to trigger oxidative stress. Research has identified an imbalance between enzymes that generate reactive oxygen species (ROS) and antioxidants in pre-eclamptic placentas (41, 42). Furthermore, studies indicate that heme oxygenase (HO), a catalyst for heme degradation, plays a crucial role in maternal and fetal vascular function, as well as placental development and function (43, 44).

The contemporary management of PE in developed countries involves a multifaceted approach encompassing pre-pregnancy counseling, perinatal blood pressure monitoring and control, antenatal administration of aspirin for high-risk women, betamethasone for patients less than 34 weeks gestation, intravenous magnesium sulfate, and meticulous postpartum blood pressure monitoring (23). The prompt delivery of the fetus and placenta remains the sole definitive treatment (1). The considerable success achieved with small molecules in treating hypertensive disorders of pregnancy offers novel therapeutic avenues for the treatment of PE.

## 3 Single-cell RNA sequencing

Tang et al. (45) first reported a sequencing technology capable of achieving high-efficiency, unbiased sequencing of cDNA up to 3,000 base pairs (kb) within a single cell. This technology is regarded as the first relatively complete generation of scRNA-seq technology.

In recent years, single-cell transcriptomics has achieved significant technological breakthroughs, with costs being reduced markedly while automation and throughput have increased substantially. scRNA-seq is a technique that allows for the analysis of transcriptomes at the single-cell level, thereby enabling the study of millions of cells in a single experiment. This methodology facilitates the classification, characterization and distinction of individual cells at the transcriptional level, thereby enabling the identification of rare yet functionally significant cell populations (17, 46, 47).

Single-cell RNA sequencing offers the advantages of high resolution, high sensitivity, and the ability to dynamically monitor transcriptional profiles, making it particularly suitable for analyzing tissues that are difficult to dissociate (48, 49) or samples containing cells with irregular sizes and shapes (50). The workflow of the method is comprised of the following stages: single-cell isolation and capture, reverse transcription and cDNA amplification, establishment of single-cell libraries, high-throughput sequencing, and data analysis (17, 51). The process of cell separation may be achieved through a variety of methodologies, including the isolation of entire cells, cell-specific nuclei, or cell-specific organelles. Alternatively, the isolation of desired cells expressing specific marker proteins can be employed. The ability to swiftly, precisely and efficiently capture individual cells continues to represent a significant challenge in the realm of single-cell sequencing (52, 53). Conventional microscopy techniques have been utilized to capture individual cells from low-abundance samples; however, this approach is laborious and has a low throughput capacity. The methodology employed for the isolation and capture of individual cells varies considerably depending on the organism, the nature of the tissue, or the characteristics of the cells in question (54). Conventional single-cell isolation and capture techniques encompass limiting dilution, fluorescence-activated cell sorting (FACS), and laser microdissection (LCM) (17). Recent technological advancements have resulted in a notable enhancement in the efficiency, scale, and precision of single-cell isolation, primarily through the utilization of cell sorting platforms based on magnetic-activated cell sorting (MACS) and microfluidics. These methodologies are now the preferred approach for single-cell separation on transcriptomic platforms (55–58).

The process of cDNA synthesis and polyadenylation of RNA enrichment is both achieved through reverse transcription. However, it is estimated that only 10%–20% of transcripts undergo reverse transcription (59). This step is critical, given the highly dependent nature of scRNA-seq efficiency. With the exception of the primer-designed RNA sequencing method DP-Seq (60), all published studies employ poly(T) primer approaches (61–63).

Second-strand synthesis in reverse transcription can be achieved via polyadenylation tailing (64) or template switching mechanisms and STRT-Seq technology (63). The former method is rapid, but it introduces amplification errors, with termination of the reverse transcriptase reaction reducing coverage at the 5′ end of transcripts. The latter method’s primary benefit is its capacity to obtain full-length transcript coverage, thus reducing 3′ end coverage bias caused by incomplete reverse transcription. However, it is important to note that the sensitivity of this method is lower than that of the previously mentioned method (62, 65).

Subsequent to the conversion of RNA into the first strand of cDNA, the resulting cDNA is amplified via polymerase chain reaction (PCR) or *in vitro* transcription (IVT) (66). PCR is a non-linear amplification process, achieved through two techniques. The first of these is SMART technology, the most commonly employed method for cDNA amplification (67). The alternative technique involves the ligation of poly(A) or poly(C) to the 5′ end of cDNA, thus constructing universal adapters for PCR reactions (17). IVT is a linear amplification process that necessitates an additional round of reverse transcription on amplified RNA, thereby introducing further 3′ coverage bias (17). It is important to note that both of these methods have the potential to induce amplification bias. In order to overcome amplification-related biases, unique molecular identifiers (UMIs) were incorporated during the reverse transcription step, thus enabling the barcoding of individual mRNA molecules within a cell. This enhanced the quantitative nature of scRNA-seq and improved read accuracy by effectively eliminating PCR amplification bias (68).

Once single-cell barcoded cDNA has been generated from a single cell or mononuclear cell, it can be sequenced using numerous deep sequencing platforms. In high-throughput sequencing based on DNA nanoballs (DNBseq), selected DNA fragments are repaired to blunt ends and modified at all three ends to incorporate dATP overhangs. Subsequently, dTTP tailing sequences are ligated to each end of the DNA fragments. Subsequent to this, the ligation products are amplified through several cycles prior to undergoing the next single-strand cycle. A specific strand of the PCR product undergoes reverse complementary ligation with a specific molecule, which is subsequently joined to the single-stranded molecule via DNA ligase, ultimately yielding a single-stranded circular DNA library (17).

**FIGURE 1:**
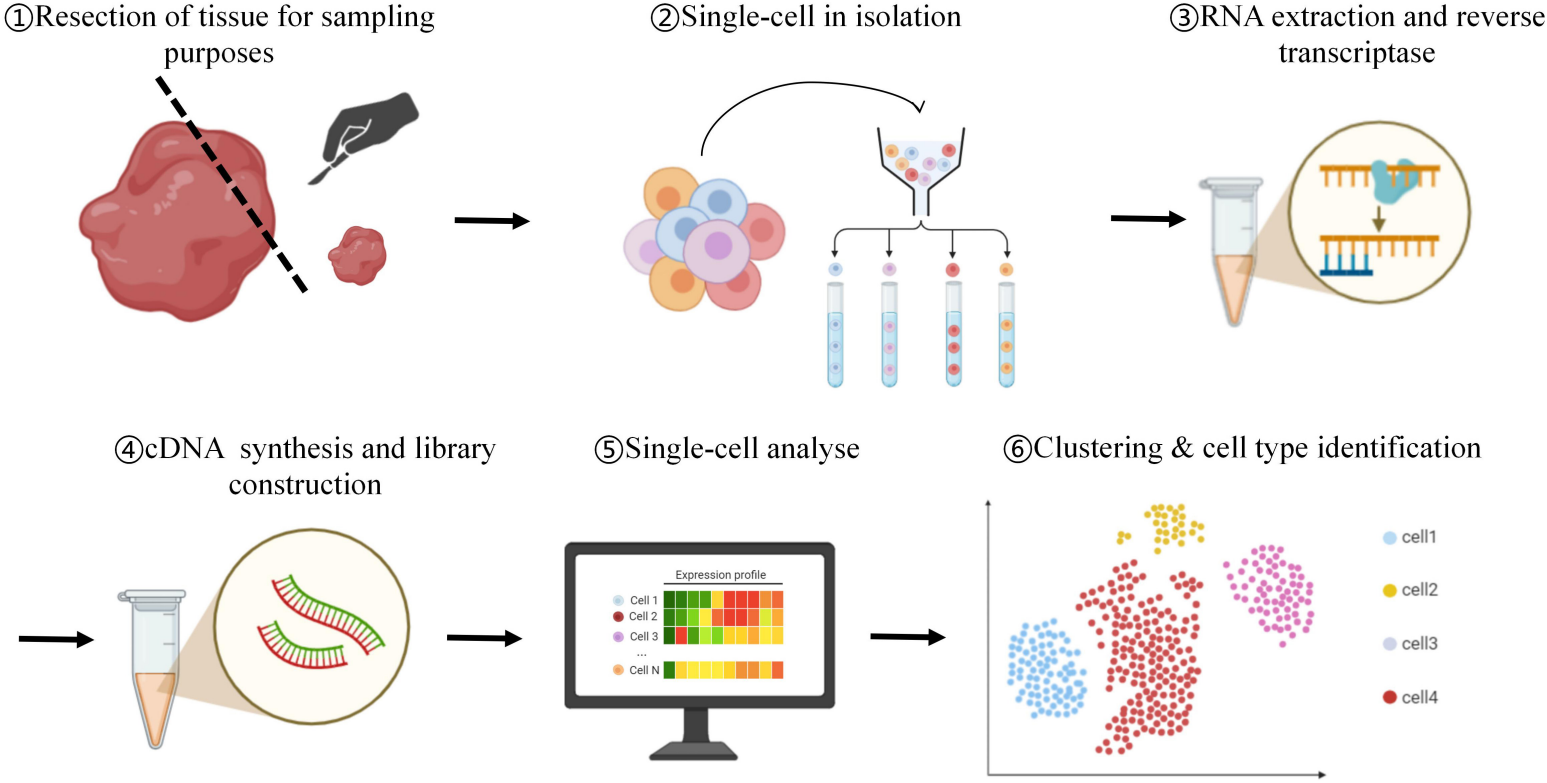
Schematic overview of the single-cell RNA-seq workflow. ➀ Tissue resection and sampling: tissue is dissected and collected for subsequent single-cell dissociation. ➁ Single-cell isolation: individual cells are separated using techniques such as microfluidics or FACS. ➂ RNA extraction and reverse transcription: RNA is extracted from each isolated cell and reverse-transcribed into complementary DNA (cDNA). ➃ cDNA synthesis and library construction: the cDNA is amplified and converted into a sequencing library suitable for high-throughput sequencing. ➄ Single-cell analysis: sequencing data are processed and quantified to generate gene expression profiles for individual cells. ➅ Clustering and cell type identification: unsupervised clustering and marker gene analysis are performed to classify cells into distinct populations and assign putative cell identities.

The analysis of scRNA-seq data involves a series of procedures, including the alignment of sequencing fragments, the implementation of quality control (QC) measures, quantification, data normalization, the removal of confounding factors, dimensionality reduction, feature selection, clustering analysis, and downstream data interpretation (1) (Figure 1).

As a crucial method for revealing cellular metabolites and expressed genes, scRNA-seq is primarily employed to analyze interactions between cellular subpopulations and gene regulatory processes (69). Its application is currently extensive, encompassing diverse research domains, including embryonic development (70, 71), tissue and organ development (72), and studies of tumors and the immune system (73).

## 4 scRNA-seq and the mechanisms of PE

Campbell et al. (74) collected villous placental tissue biopsies from placentas shortly after delivery from healthy, full-term, singleton uncomplicated Cesarean sections. After cutting away the basal and chorionic plates and scraping the villous tissue, they performed dissociation to generate single-cell RNA sequencing samples. By integrating these samples with existing data, they established the largest deconvolution reference to date, encompassing 19 fetal and 8 maternal cell types. Their findings revealed that significant placental cellular heterogeneity in preeclampsia contributes to the numerous previously observed gene expression differences, laying the foundation for studies investigating the role of cellular heterogeneity in placental dysfunction and adverse birth outcomes. Muñoz-Blat et al. (75), through scRNA-seq of endometrial samples from patients with severe preeclampsia (sPE), revealed a cellular composition characterized by a stromal mosaic state, featuring proliferating stromal cells (MMP11^+^ and SFRP4^+^) alongside IGFBP1^+^ decidualized cells, concurrent with an epithelial mosaic and an absence of epithelial–stromal transformation associated with decidualization.

Early-onset and late-onset preeclampsia exhibit distinctly different cellular pathological features. Solt et al. (76), through single-cell and single-nucleus RNA sequencing analysis of approximately 90,000 placental cells, found that early-onset preeclampsia (EOPE) is characterized by widespread dysregulation across multiple cell lineages, including disrupted angiogenic signaling pathways in syncytiotrophoblasts and extravillous trophoblasts (upregulation of FLT1, downregulation of PGF), stress responses in stromal and vascular compartments, and significant upregulation of pro-inflammatory cytokines (e.g., SPP1, CXCL2, CXCL3) in maternal-fetal immune cells. In contrast, late-onset preeclampsia (LOPE) only exhibited focal dysregulation of extracellular matrix remodeling and angiogenesis markers, retaining certain features of placental adaptability.

Immune cell dysfunction is considered one of the mechanisms of PE. Multiple studies point to the roles of macrophages, NK cells, and T cells in the pathogenesis of PE. Fei et al. (77) demonstrated that transferring F4/80^+^CD206^+^ pro-inflammatory monocytes with a Folr2^+^Ccl7^+^Ccl8^+^C1qa^+^C1qb^+^C1qc^+^ phenotype from the uteri of PE-like mouse models into normal pregnant mice could promote the generation of memory-like Th17 cells via the IGF1-IGF1R pathway, thereby inducing the occurrence and recurrence of preeclampsia-like symptoms. This study revealed a PE-specific immune cell network regulated by pro-inflammatory monocytes, providing new insights into the pathogenesis of the disease. Uterine natural killer (uNK) cells are thought to regulate trophoblast invasion and spiral artery remodeling (78). Research by Whettlock et al. (79) also revealed that uNK cells can be divided into three functionally distinct subsets, playing key roles in embryo implantation and spiral artery remodeling. Xiao et al. (80), using scRNA-seq and Gene Set Variation Analysis (GSVA) on placental and decidual tissues for differential expression, cell reclustering, and pseudotime analysis, discovered that understanding how T cells differentiate during early pregnancy and how the CD8^+^ T-cell-dominated immune environment affects villous cytotrophoblast (VCT) differentiation is crucial for elucidating the pathogenesis of PE. Rong et al. (81) downloaded raw single-cell sequencing data and gene expression matrices from the Gene Expression Omnibus (GEO) and found that aberrant gene expression affects the efferocytosis function of decidual macrophages, subsequently impacting their interaction with other immune cells, thereby disrupting the original immune regulatory mechanism and ultimately leading to the occurrence of preeclampsia. Couture et al. (82) have demonstrated that placental CD163^+^ cells and 1st trimester blood pressures can be valuable non-invasive and predictive biomarkers of PPPE with strong clinical application prospects. Notably, immune cells in early-onset preeclampsia exhibit a more pronounced pro-inflammatory activation state, whereas immune alterations in late-onset preeclampsia are relatively limited (76) (Table 1).

**TABLE 1:** Summary of representative studies applying single-cell RNA sequencing (scRNA-seq) to investigate preeclampsia (PE).

Data set	Sample source	Tissue type	Time	Key findings
Campbell et al. (74)	Healthy, singleton, uncomplicated pregnant women	Placenta	After term Cesarean section	Generated single-cell RNA sequencing samples, integrated with existing data to create the largest deconvolution reference for 19 fetal and 8 maternal cell types.
Muñoz-Blat et al. (75)	Non-pregnant women: with a history of previous severe preeclampsia (sPE) pregnancy and with a history of previous normal pregnancy	Endometrial	Late secretory phase of the menstrual cycle	Revealed DR cell and molecular characteristics associated with former sPE patients.
Liu et al. (84)	PE patients, mouse models	Placenta	/	CD74 plays an important protective role in the pathogenesis of PE by regulating the MAPK signaling pathway, which can be regulated by SIRT1.
He et al. (85)	Pregnant mice (PE-like group, normal control group)	Cell types expressing NEP in the placenta	/	NEP, as a protein cargo in PL-EVs, links the two developmental stages of preeclampsia by disrupting endothelial CNP-dependent VSMC relaxation, thereby contributing to the clinical manifestations of preeclampsia.
Liu et al. (88)	EOPE group, normal control group	Placental and decidual tissue	After term pregnancy Cesarean section	The expression of DAB2 in EVT is crucial for coordinating the phenotypic switch and motility of dVSMCs. These processes may be closely related to the CXCL8/PI3K/AKT pathway, highlighting its core role in complex SPA remodeling.
Botha et al. (86)	EOPE group, normal control group	Maternal plasma	36 weeks of gestation	Cystatin 6 (CST6) and Legumain (LGMN) are potential mediators in the pathogenesis of PE.
		Placenta	Within 30 min after term delivery	
Xiao et al. (80)	PE group, normal control group	Placental and decidual tissue	After term pregnancy Cesarean section	Revealed cellular dysfunction at the maternal-fetal interface in preeclampsia patients.
Rong et al. (81)	PE group, normal control group	Gene Expression Omnibus	/	Dysfunction of decidual macrophages is a potential risk factor in the occurrence of PE.
Fei et al. (77)	PE patients, mouse models	Uterus from pregnant mice and placentas from volunteers	/	Revealed the PE-specific immune cell network, which was regulated by pro-inflam Macs, providing new ideas about the pathogenesis of PE.
Jiang et al. (83)	PE group, normal control group	Placenta	Within 10 min after term pregnancy Cesarean section	Identified JUNB as a key gene in macrophages within PE; interfering with JUNB expression can promote macrophage polarization toward the M2 phenotype. Research indicates JUNB affects PE through the MIIP/PI3K/AKT pathway.
Huang et al. (93)	PE group, normal control group	Peripheral venous blood	11^+6^–13 weeks of gestation, 4–7 days before delivery	A2M is a potential target for EOPE, providing initial therapy for inhibiting the A2M-low density lipoprotein receptor-related protein 1 (LRP1) combination.
Li et al. (92)	PE group, normal control group	Decidual tissue	6–8 weeks of gestation, late pregnancy	KIR^+^CD8^+^ T cells increase during human pregnancy, potentially promoting maternal tolerance by inhibiting fetal-specific T cells.
		Human peripheral blood and peripheral blood mononuclear cell (PBMC)	First trimester, second trimester, third trimester	

In this table summarizes key studies cited in this review, detailing data sources, sample sources, tissue types, timing of sample collection, and major findings related to PE pathogenesis, immune dysregulation, and therapeutic targets. EOPE, early-onset preeclampsia; EVT, extravillous trophoblast; dVSMC, decidual vascular smooth muscle cell; NEP, neprilysin; PL-EVs, placental extracellular vesicles; VSMC, vascular smooth muscle cell; SPA, spiral artery; CST6, cystatin 6; LGMN, legumain; A2M, alpha-2-macroglobulin; LRP1, low-density lipoprotein receptor-related protein 1; KIR, killer-cell immunoglobulin-like receptor; PBMC, peripheral blood mononuclear cell; “/” indicates not reported or not applicable.

Dysregulation of specific signaling pathways has been validated across multiple independent studies. For example, Solt et al. (76) confirmed the prevalence of FLT1/PGF imbalance in early-onset preeclampsia. Jiang et al. (83) revealed a novel mechanism regulating macrophage polarization through the JUNB-MIIP-PI3K/AKT axis. Liu et al. (84) discovered through scRNA-seq technology that the MAPK signaling pathway, which can be regulated by SIRT1, plays an important protective role in the pathogenesis of PE. He et al. (85) intravenously injected extracellular vesicles (EVs) from normal placentas and those complicated by preeclampsia into pregnant mice and recorded blood pressure throughout gestation. They found that PE placental EVs (PE-PL-EVs) induced preeclampsia-like clinical manifestations in pregnant mice, including hypertension, proteinuria, and fetal growth restriction. Botha et al. (86), through computational analysis of two publicly available single-cell and single-nucleus RNA sequencing datasets, discovered an inverse relationship between CST6 and LGMN in the placenta and the maternal circulation in preeclampsia. They thus proposed that elevated circulating levels of CST6 might be caused by placental hypoxia. Cysteine-rich angiogenic inducer 61 (CYR61), also known as CCN1, is widely distributed throughout the body, particularly in vascular-rich tissues, developing embryonic structures, and tissues requiring repair. Previous studies have reported that CCN1 promotes cell adhesion, proliferation, migration, and angiogenesis. Analysis of public single-cell RNA sequencing datasets revealed that CYR61 expression is significantly downregulated in EVT cells of preeclamptic placentas. The G protein-coupled estrogen receptor (GPER) is expressed in human EVT cells and has been linked to promoting cell invasion. Wang et al. (87) identified a novel GPER-YAP-Snail-CYR61 signaling axis that regulates EVT invasion and suggested that dysregulation of this pathway may contribute to defective trophoblast invasion in PE. Liu et al. (88) performed single-cell RNA sequencing analysis on whole placental tissues from patients with early-onset preeclampsia (EOPE) and their corresponding controls. From genes including DAB2, MMP2, MMP9, α-SMA, SM22α, Calponin, MYH11, GAPDH, IL20R, CCL20, IL1A, CXCL2, CXCL1, CXCL6, CXCL3, TGFA, and IL21R, they selected DAB2 as the key gene of interest. Through cell models and a placenta-decidua co-culture (PDC) model, they explored the mechanisms of communication between extravillous trophoblasts (EVT) and decidual vascular smooth muscle cells (dVSMC) *in vitro*, discovering the core role of DAB2 in complex spiral artery remodeling.

Notably, several genes closely associated with the pathogenesis of PE are genomically imprinted genes, whose expression is tightly regulated by epigenetic mechanisms such as DNA methylation (89). For example, IGF2 (insulin-like growth factor 2) and H19 are a classic imprinted gene pair that play central roles in placental development and fetal growth. Loss of imprinting of IGF2/H19 in trophoblast cells may lead to overexpression or silencing of IGF2, thereby affecting placental nutrient delivery and vascular remodeling (90). In addition, imprinted genes such as CDKN1C (p57KIP2), PEG10, and PLAGL1 are highly expressed in placental trophoblast cells and participate in cell proliferation, differentiation, and vascular remodeling. Among these, aberrant methylation of CDKN1C may interfere with the decidualization process and contribute to the pathogenesis of PE (91) (Table 1).

## 5 scRNA-seq and the treatment of PE

Single-cell RNA sequencing has not only deepened the understanding of PE pathogenesis but also provided clues for the discovery of therapeutic targets.

Targeting the polarization state of immune cells may serve as a therapeutic approach for PE. Jiang et al. (83) utilized single-cell RNA sequencing (scRNA-seq) to delineate the cellular landscape of the placenta in PE, identifying 11 distinct cell subsets, among which macrophages played a key role in mediating intercellular communication. They discovered that the transcription factor JUNB is a key gene in macrophages, influencing the interactions between macrophages and epithelial as well as endothelial cells. Interference with JUNB expression promoted macrophage polarization toward the M2 phenotype, thereby facilitating trophoblast invasion, migration, and angiogenesis. The study further demonstrated that targeting JUNB could activate the PI3K/AKT pathway via transcriptional activation of MIIP, thereby promoting M2 polarization and potentially delaying the onset of PE. Similarly, Li et al. (92), through analysis of peripheral blood and peripheral blood mononuclear cells (PBMCs) from pregnant women, found that KIR^+^CD8^+^ T cells may promote maternal tolerance by regulating fetal-specific alloreactive T cell responses, and that higher frequencies of KIR^+^CD8^+^ T cells are associated with preeclampsia.

Intervening in specific signaling pathways is also a research direction. Huang et al. (93), through scRNA-seq analysis of peripheral venous blood samples, placental, and decidual tissues from pregnant women, found that smooth muscle cell (SMC) α2-macroglobulin (A2M) promotes the progression of preeclampsia by directly upregulating RhoA-GTPase. Their results also indicated that A2M is a potential target for EOPE, providing initial therapeutic insights for inhibiting the A2M-low density lipoprotein receptor-related protein 1 (LRP1) combination. Research by Huang et al. (94) found that c-Fos silencing inhibits p-AMPK (phosphorylated AMP-activated protein kinase)/deacetylated tubulin pathway, disrupting lipid droplet transport and metabolism, promoting lipid droplet accumulation and altered lipid profiles. c-Fos deficiency induced a preeclampsia-like phenotype in mice, while c-Fos overexpression partially alleviated preeclampsia symptoms. This suggests that c-Fos is a potential therapeutic target for preeclampsia.

Many studies explore novel therapeutic approaches for PE by identifying key genes. Zhong et al. (95) integrated preeclampsia-specific genome-wide association study (GWAS) data, expression and protein quantitative trait loci (eQTL and pQTL) data, as well as single-cell data from peripheral blood mononuclear cells (PBMCs). Using scRNA-seq technology, they identified and highlighted significant cellular differences, determining IFITM3, NINJ1, COTL1, CD69, and YWHAZ as key genes associated with PE pathogenesis. Ruan et al. (96) utilized scRNA-seq to identify shared hub genes and immune pathways in PE, characterized by crosstalk between BIN2, LYN, NEDD9, and PIK3AP1, shedding light on the pathogenesis of PE, which may pave the way for developing effective diagnostic, therapeutic, and management strategies.

Early-onset and late-onset preeclampsia may require specific therapeutic strategies. Based on single-cell atlases, Solt et al. (76) proposed that early-onset preeclampsia could benefit from interventions targeting angiogenic pathways, whereas the management of late-onset preeclampsia should focus on optimizing maternal cardiovascular function. Research by Bernier et al. (97) further confirmed significant differences in immune cell profiles between early-onset and late-onset preeclampsia patients, as well as distinct effects on the vascular endothelium, suggesting that treatment strategies must consider subtype specificity. Zheng et al. (98) discovered that HSD17B1 is a major coordinator of trophoblast-endocrine crosstalk, and that impaired trophoblast crosstalk in placental trophoblasts may contribute to EOPE pathogenesis. This provides a mechanistic basis for developing HSD17B1-targeted interventions that may help simultaneously restore placental capacity and hormonal regulation, thereby improving perinatal outcomes in EOPE patients.

## 6 scRNA-seq and the diagnosis and risk prediction of PE

Over the past 30 years, the incidence of gestational hypertension and preeclampsia has increased alarmingly, posing a serious threat to the life safety of pregnant women. Therefore, early prediction of risk and assessment of patient prognosis are of paramount importance (99).

Polygenic combination models are considered a tool for PE diagnosis and risk prediction. Xiong et al. (100), by integrating WGCNA and machine learning algorithms, screened five characteristic genes from 101 metabolism-related genes to construct a predictive model, enhancing predictive efficacy. Four apoptosis-related genes identified by Liu et al. (101)–CD44, MIF, PIK3R1, and TLR4–were also integrated into a diagnostic nomogram, demonstrating good clinical utility. Tarca et al. (102) found that placenta cell-specific mRNAs in maternal peripheral blood, such as H19, FN1, TUBB6, and FPR3, could predict the future development of PE, offering new insights for non-invasive early screening. Sun et al. collected placental and plasma samples from pregnant women to detect TPBG (trophoblast glycoprotein) expression and explored the downstream molecular mechanisms of TPBG using RNA sequencing and single-cell RNA sequencing data. They discovered that TPBG overexpression increases the risk of preeclampsia through its disruptive effects on trophoblast and extravillous trophoblast migration/invasion during spiral artery remodeling; consequently, TPBG levels in maternal blood may serve as a predictor of PE risk (103).

Different PE subtypes possess specific markers. Guo et al. (104) systematically compared the transcriptomic features of early-onset and late-onset PE, identifying subtype-specific biomarkers such as EBI3, IGF2, ORMDL3, GATA2, and KIR2DL4, and validated them at the protein level. Liu et al. (101) also found that CD44, MIF, and PIK3R1 exhibited opposing expression trends in early-onset versus late-onset PE (downregulated in early-onset, upregulated in late-onset), reflecting the placenta’s adaptive response to different adverse pregnancy conditions (Table 1).

## 7 Conclusion and prospect

Recent years have seen a major advancement in our understanding of the cellular composition of complex organs in health and disease through the application of scRNA-seq (105–107). This technological advancement has also facilitated a more profound comprehension of cellular diversity and heterogeneity across diverse tissuing (16, 108, 109), including the placenta (22, 110–114). scRNA-seq enables a comprehensive analysis of gene expression, thus enabling the cataloging of cell types and the identification of molecular dysregulation associated with perturbations or disease at the level of individual cells.

In the context of placental and PE research, scRNA-seq offers the following advantages: Firstly, in contrast to batch RNA-seq, scRNA-seq is capable of addressing cellular heterogeneity at the maternal-fetal interface, thus providing a distinct advantage for the study of the placenta in PE. Secondly, scRNA-seq has the capacity to identify alterations in gene expression, including temporal variations, at the single-cell type level (e.g., trophoblasts, endothelial cells, immune cells, and stromal cells). This process enables the determination of their specific contributions to the pathogenesis of PE and the identification of potential pathways for targeted therapies. Thirdly, scRNA-seq has the capacity to detect rare cell populations that are potentially pivotal in the context of PE pathology, such as aberrant stromal cells or inflammatory cells. Such cell populations may be obscured in batch analyses. Fourthly, scRNA-seq facilitates analysis of immune cell dynamics, trophoblast invasion defects, endothelial dysfunction, and disrupted intercellular communication, thereby aiding exploration of angiogenesis mechanisms.

Single-cell RNA sequencing is also subject to certain limitations, including the accessibility of samples (e.g., the procurement of placental or decidual tissue) and the cost and complexity of the technique itself. Furthermore, scRNA-seq faces challenges in addressing the multinucleated structure of syncytiotrophoblasts, necessitating the employment of single-nucleus RNA-seq technologies to overcome this shortcoming (1, 115–118).

This review systematically elaborates on the significant role of single-cell RNA sequencing (scRNA-seq) in preeclampsia (PE) research and distills the following core perspectives through integrative analysis: First, early-onset and late-onset PE exhibit fundamentally distinct cytopathological features, with the former characterized by multi-lineage placental cell dysfunction and the latter by relatively localized molecular alterations. Second, dysregulation of the immune microenvironment is a common pathway in PE, though its manifestations are subtype-specific. Third, angiogenic imbalance is a mechanism validated across multiple cross-validated studies. Fourth, signaling pathways such as PI3K/AKT and MAPK have been identified as key regulatory nodes with potential as therapeutic targets. Fifth, polygenic combination-based predictive models and liquid biopsy technologies offer new avenues for the early diagnosis and risk prediction of PE.

However, current research still faces numerous challenges, including a lack of unified criteria for defining cell subpopulations, limited overlap in key genes across studies, heterogeneity in results arising from differences in technological platforms and methodologies, and bottlenecks in translating mechanistic discoveries into clinical applications. Most findings lack validation in large-scale prospective cohorts, and functional experiments alongside clinical translational research urgently need to be strengthened. Consequently, further in-depth investigation is required. Looking ahead, the future holds promise for offering highly promising therapeutic strategies for patients with PE.

## References

[1] Rana S Lemoine E Granger JP Karumanchi SA. Preeclampsia: pathophysiology, challenges, and perspectives. *Circ Res.* (2019) 124:1094–112. 30920918 10.1161/CIRCRESAHA.118.313276

[2] Erez O Romero R Jung E Chaemsaithong P Bosco M Suksai M et al. Preeclampsia and eclampsia: the conceptual evolution of a syndrome. *Am J Obstet Gynecol.* (2022) 226:S786–803. 10.1016/j.ajog.2021.12.001 35177220 PMC8941666

[3] Wallis AB Saftlas AF Hsia J Atrash HK. Secular trends in the rates of preeclampsia, eclampsia, and gestational hypertension, United States, 1987-2004. *Am J Hypertens.* (2008) 21:521–6. 10.1038/ajh.2008.20 18437143

[4] Duley L. The global impact of pre-eclampsia and eclampsia. *Semin Perinatol.* (2009) 33:130–7. 10.1053/j.semperi.2009.02.010 19464502

[5] Brown MA Magee LA Kenny LC Karumanchi SA McCarthy FP Saito S et al. Hypertensive disorders of pregnancy: ISSHP classification, diagnosis, and management recommendations for international practice. *Hypertension.* (2018) 72:24–43. 10.1161/hypertensionaha.117.10803 29899139

[6] Redman C Staff AC Roberts JM. Syncytiotrophoblast stress in preeclampsia: the convergence point for multiple pathways. *Am J Obstet Gynecol.* (2022) 226:S907–27. 10.1016/j.ajog.2020.09.047 33546842

[7] Brosens I Pijnenborg R Vercruysse L Romero R. The “Great Obstetrical Syndromes” are associated with disorders of deep placentation. *Am J Obstet Gynecol.* (2011) 204:193–201. 10.1016/j.ajog.2010.08.009 21094932 PMC3369813

[8] Goldman-Wohl D Yagel S. Regulation of trophoblast invasion: from normal implantation to pre-eclampsia. *Mol Cell Endocrinol.* (2002) 187:233–8. 10.1016/s0303-7207(01)00687-6 11988332

[9] Egbor M Ansari T Morris N Green CJ Sibbons PD. Morphometric placental villous and vascular abnormalities in early- and late-onset pre-eclampsia with and without fetal growth restriction. *BJOG.* (2006) 113:580–9. 10.1111/j.1471-0528.2006.00882.x 16579806

[10] Staff AC Redman CW Williams D Leeson P Moe K Thilaganathan B et al. Pregnancy and long-term maternal cardiovascular health: progress through harmonization of research cohorts and biobanks. *Hypertension.* (2016) 67:251–60. 10.1161/hypertensionaha.115.06357 26667417

[11] Yagel S Cohen SM Goldman-Wohl D. An integrated model of preeclampsia: a multifaceted syndrome of the maternal cardiovascular-placental-fetal array. *Am J Obstet Gynecol.* (2022) 226:S963–72. 10.1016/j.ajog.2020.10.023 33712272

[12] Yuan Q Lv N Chen Q Shen S Wang Y Tong J. Application of single cell sequencing technology in ovarian cancer research (review). *Funct Integr Genomics.* (2024) 24:144. 10.1007/s10142-024-01432-w 39196391 PMC11358195

[13] Kashima Y Sakamoto Y Kaneko K Seki M Suzuki Y Suzuki A. Single-cell sequencing techniques from individual to multiomics analyses. *Exp Mol Med.* (2020) 52:1419–27. 10.1038/s12276-020-00499-2 32929221 PMC8080663

[14] Li Y Ma L Wu D Chen G. Advances in bulk and single-cell multi-omics approaches for systems biology and precision medicine. *Brief Bioinform.* (2021) 22:bbab024. 10.1093/bib/bbab024 33778867

[15] Shu C Street K Breton CV Bastain TM Wilson ML. A review of single-cell transcriptomics and epigenomics studies in maternal and child health. *Epigenomics.* (2024) 16:775–93. 10.1080/17501911.2024.2343276 38709139 PMC11318716

[16] Haque A Engel J Teichmann SA Lönnberg T. A practical guide to single-cell RNA-sequencing for biomedical research and clinical applications. *Genome Med.* (2017) 9:75. 10.1186/s13073-017-0467-4 28821273 PMC5561556

[17] Jovic D Liang X Zeng H Lin L Xu F Luo Y. Single-cell RNA sequencing technologies and applications: a brief overview. *Clin Transl Med.* (2022) 12:e694. 10.1002/ctm2.694 35352511 PMC8964935

[18] Potter SS. Single-cell RNA sequencing for the study of development, physiology and disease. *Nat Rev Nephrol.* (2018) 14:479–92. 10.1038/s41581-018-0021-7 29789704 PMC6070143

[19] Walter TJ Suter RK Ayad NG. An overview of human single-cell RNA sequencing studies in neurobiological disease. *Neurobiol Dis.* (2023) 184:106201. 10.1016/j.nbd.2023.106201 37321420 PMC10470823

[20] Zhang T Bian Q Chen Y Wang X Yu S Liu S et al. Dissecting human trophoblast cell transcriptional heterogeneity in preeclampsia using single-cell RNA sequencing. *Mol Genet Genomic Med.* (2021) 9:e1730. 10.1002/mgg3.1730 34212522 PMC8404237

[21] Zhou W Wang H Yang Y Guo F Yu B Su Z. Trophoblast cell subtypes and dysfunction in the placenta of individuals with preeclampsia revealed by single-cell RNA sequencing. *Mol Cells.* (2022) 45:317–28. 10.14348/molcells.2021.0211 35289305 PMC9095508

[22] Yang J Gong L Liu Q Zhao H Wang Z Li X et al. Single-cell RNA-seq reveals developmental deficiencies in both the placentation and the decidualization in women with late-onset preeclampsia. *Front Immunol.* (2023) 14:1142273. 10.3389/fimmu.2023.1142273 37283740 PMC10239844

[23] Hypertension in Pregnancy. Report of the American College of Obstetricians and gynecologists’ task force on hypertension in pregnancy. *Obstet Gynecol.* (2013) 122:1122–31. 10.1097/01.AOG.0000437382.03963.88 24150027

[24] Hogan MC Foreman KJ Naghavi M Ahn SY Wang M Makela SM et al. Maternal mortality for 181 countries, 1980-2008: a systematic analysis of progress towards Millennium Development Goal 5. *Lancet.* (2010) 375:1609–23. 10.1016/s0140-6736(10)60518-1 20382417

[25] Malhamé I Nerenberg K McLaughlin K Grandi SM Daskalopoulou SS Metcalfe A. Hypertensive disorders and cardiovascular severe maternal morbidity in the US, 2015-2019. *JAMA Netw Open.* (2024) 7:e2436478. 10.1001/jamanetworkopen.2024.36478 39361284 PMC11581633

[26] Wanderer JP Leffert LR Mhyre JM Kuklina EV Callaghan WM Bateman BT. Epidemiology of obstetric-related ICU admissions in Maryland: 1999-2008*. *Crit Care Med.* (2013) 41:1844–52. 10.1097/ccm.0b013e31828a3e24 23648568 PMC3716838

[27] Goel A Maski MR Bajracharya S Wenger JB Zhang D Salahuddin S et al. Epidemiology and mechanisms of de novo and persistent hypertension in the postpartum period. *Circulation.* (2015) 132:1726–33. 10.1161/circulationaha.115.015721 26416810 PMC4816491

[28] Bernstein PS Martin JN Jr. Barton JR Shields LE Druzin ML Scavone BM et al. National partnership for maternal safety: consensus bundle on severe hypertension during pregnancy and the postpartum period. *Obstet Gynecol.* (2017) 130:347–57. 10.1097/aog.0000000000002115 28697093

[29] Coutinho T Lamai O Nerenberg K. Hypertensive disorders of pregnancy and cardiovascular diseases: current knowledge and future directions. *Curr Treat Options Cardiovasc Med.* (2018) 20:56. 10.1007/s11936-018-0653-8 29923067

[30] Pankiewicz K Fijałkowska A Issat T Maciejewski TM. Insight into the key points of preeclampsia pathophysiology: uterine artery remodeling and the role of microRNAs. *Int J Mol Sci.* (2021) 22:3132. 10.3390/ijms22063132 33808559 PMC8003365

[31] Staff AC. The two-stage placental model of preeclampsia: an update. *J Reprod Immunol.* (2019) 13:1–10. 10.1016/j.jri.2019.07.004 31301487

[32] Sato Y Fujiwara H Konishi I. Mechanism of maternal vascular remodeling during human pregnancy. *Reprod Med Biol.* (2012) 11:27–36. 10.1007/s12522-011-0102-9 29699103 PMC5906868

[33] Chiang YT Seow KM Chen KH. The pathophysiological, genetic, and hormonal changes in preeclampsia: a systematic review of the molecular mechanisms. *Int J Mol Sci.* (2024) 25:4532. 10.3390/ijms25084532 38674114 PMC11050545

[34] Kornacki J Olejniczak O Sibiak R Gutaj P Wender-Ożegowska E. Pathophysiology of pre-eclampsia-two theories of the development of the disease. *Int J Mol Sci.* (2023) 25:307. 10.3390/ijms25010307 38203478 PMC10779413

[35] Zhou Y Damsky CH Fisher SJ. Preeclampsia is associated with failure of human cytotrophoblasts to mimic a vascular adhesion phenotype. One cause of defective endovascular invasion in this syndrome. *J Clin Invest.* (1997) 99:2152–64. 10.1172/jci119388 9151787 PMC508045

[36] Brosens JJ Parker MG McIndoe A Pijnenborg R Brosens IA. A role for menstruation in preconditioning the uterus for successful pregnancy. *Am J Obstet Gynecol.* (2009) 200:615.e1–6. 10.1016/j.ajog.2008.11.037 19136085

[37] Garrido-Gomez T Dominguez F Quiñonero A Diaz-Gimeno P Kapidzic M Gormley M et al. Defective decidualization during and after severe preeclampsia reveals a possible maternal contribution to the etiology. *Proc Natl Acad Sci U S A.* (2017) 114:E8468–77. 10.1073/pnas.1706546114 28923940 PMC5635883

[38] Gray KJ Saxena R Karumanchi SA. Genetic predisposition to preeclampsia is conferred by fetal DNA variants near FLT1, a gene involved in the regulation of angiogenesis. *Am J Obstet Gynecol.* (2018) 218:211–8. 10.1016/j.ajog.2017.11.562 29138037 PMC5807126

[39] Rabaglino MB Post Uiterweer ED Jeyabalan A Hogge WA Conrad KP. Bioinformatics approach reveals evidence for impaired endometrial maturation before and during early pregnancy in women who developed preeclampsia. *Hypertension.* (2015) 65:421–9. 10.1161/hypertensionaha.114.04481 25421975 PMC4290371

[40] Tal R Shaish A Barshack I Polak-Charcon S Afek A Volkov A et al. Effects of hypoxia-inducible factor-1alpha overexpression in pregnant mice: possible implications for preeclampsia and intrauterine growth restriction. *Am J Pathol.* (2010) 177:2950–62. 10.3410/f.5879960.5868058 20952590 PMC2993274

[41] Many A Hubel CA Fisher SJ Roberts JM Zhou Y. Invasive cytotrophoblasts manifest evidence of oxidative stress in preeclampsia. *Am J Pathol.* (2000) 156:321–31. 10.1016/s0002-9440(10)64733-5 10623681 PMC1868629

[42] Vaughan JE Walsh SW. Oxidative stress reproduces placental abnormalities of preeclampsia. *Hypertens Pregnancy.* (2002) 21:205–23. 10.1081/prg-120015848 12517328

[43] Cudmore M Ahmad S Al-Ani B Fujisawa T Coxall H Chudasama K et al. Negative regulation of soluble Flt-1 and soluble endoglin release by heme oxygenase-1. *Circulation.* (2007) 115:1789–97. 10.1161/circulationaha.106.660134 17389265

[44] George EM Colson D Dixon J Palei AC Granger JP. Heme oxygenase-1 attenuates hypoxia-induced sFlt-1 and oxidative stress in placental villi through its metabolic products co and bilirubin. *Int J Hypertens.* (2012) 2012:486053. 10.1155/2012/486053 22195275 PMC3238375

[45] Tang F Barbacioru C Wang Y Nordman E Lee C Xu N et al. mRNA-Seq whole-transcriptome analysis of a single cell. *Nat Methods.* (2009) 6:377–82. 10.1038/nmeth.1315 19349980

[46] Agnihotri SN Ugolini GS Sullivan MR Yang Y De Ganzó A Lim JW et al. Droplet microfluidics for functional temporal analysis and cell recovery on demand using microvalves: application in immunotherapies for cancer. *Lab Chip.* (2022) 22:3258–67. 10.1039/d2lc00435f 35904070 PMC9535857

[47] Hwang B Lee DS Tamaki W Sun Y Ogorodnikov A Hartoularos GC et al. SCITO-seq: single-cell combinatorial indexed cytometry sequencing. *Nat Methods.* (2021) 18:903–11. 10.1038/s41592-021-01222-3 34354295 PMC8643207

[48] Denisenko E Guo BB Jones M Hou R de Kock L Lassmann T et al. Systematic assessment of tissue dissociation and storage biases in single-cell and single-nucleus RNA-seq workflows. *Genome Biol.* (2020) 21:130. 10.1186/s13059-020-02048-6 32487174 PMC7265231

[49] Thomsen ER Mich JK Yao Z Hodge RD Doyle AM Jang S et al. Fixed single-cell transcriptomic characterization of human radial glial diversity. *Nat Methods.* (2016) 13:87–93. 10.1038/nmeth.3629 26524239 PMC4869711

[50] Haber AL Biton M Rogel N Herbst RH Shekhar K Smillie C et al. A single-cell survey of the small intestinal epithelium. *Nature.* (2017) 551:333–9. 10.1038/nature24489 29144463 PMC6022292

[51] de Klerk E ‘t Hoen PA. Alternative mRNA transcription, processing, and translation: insights from RNA sequencing. *Trends Genet.* (2015) 31:128–39. 10.1016/j.tig.2015.01.001 25648499

[52] Hawrylycz MJ Lein ES Guillozet-Bongaarts AL Shen EH Ng L Miller JA et al. An anatomically comprehensive atlas of the adult human brain transcriptome. *Nature.* (2012) 489:391–9. 10.3410/f.717960639.793463706 22996553 PMC4243026

[53] Li Y Wang Z Han F Zhang M Yang T Chen M et al. Single-cell transcriptome analysis profiles cellular and molecular alterations in submandibular gland and blood in IgG4-related disease. *Ann Rheum Dis.* (2023) 82:1348–58. 10.1136/ard-2023-224363 37474274

[54] Sanz E Yang L Su T Morris DR McKnight GS Amieux PS. Cell-type-specific isolation of ribosome-associated mRNA from complex tissues. *Proc Natl Acad Sci U S A.* (2009) 106:13939–44. 10.1073/pnas.0907143106 19666516 PMC2728999

[55] Han X Wang R Zhou Y Fei L Sun H Lai S et al. Mapping the mouse cell atlas by microwell-seq. *Cell.* (2018) 172:1091.e–107.e. 10.1016/j.cell.2018.02.001 29474909

[56] Valihrach L Androvic P Kubista M. Platforms for single-cell collection and analysis. *Int J Mol Sci.* (2018) 19:807. 10.3390/ijms19030807 29534489 PMC5877668

[57] Warren L Bryder D Weissman IL Quake SR. Transcription factor profiling in individual hematopoietic progenitors by digital RT-PCR. *Proc Natl Acad Sci U S A.* (2006) 103:17807–12. 10.1073/pnas.0608512103 17098862 PMC1693828

[58] Zhao XM Cui LS Hao HS Wang HY Zhao SJ Du WH et al. Transcriptome analyses of inner cell mass and trophectoderm cells isolated by magnetic-activated cell sorting from bovine blastocysts using single cell RNA-seq. *Reprod Domest Anim.* (2016) 51:726–35. 10.1111/rda.12737 27440443

[59] Islam S Zeisel A Joost S La Manno G Zajac P Kasper M et al. Quantitative single-cell RNA-seq with unique molecular identifiers. *Nat Methods.* (2014) 11:163–6. 10.1038/nmeth.2772 24363023

[60] Bhargava V Ko P Willems E Mercola M Subramaniam S. Quantitative transcriptomics using designed primer-based amplification. *Sci Rep.* (2013) 3:1740. 10.1038/srep01740 23624976 PMC3638165

[61] Hashimshony T Wagner F Sher N Yanai I. CEL-Seq: single-cell RNA-Seq by multiplexed linear amplification. *Cell Rep.* (2012) 2:666–73. 10.1016/j.celrep.2012.08.003 22939981

[62] Islam S Kjällquist U Moliner A Zajac P Fan JB Lönnerberg P et al. Characterization of the single-cell transcriptional landscape by highly multiplex RNA-seq. *Genome Res.* (2011) 21:1160–7. 10.1101/gr.110882.110 21543516 PMC3129258

[63] Ramsköld D Luo S Wang YC Li R Deng Q Faridani OR et al. Full-length mRNA-Seq from single-cell levels of RNA and individual circulating tumor cells. *Nat Biotechnol.* (2012) 30:777–82. 10.1038/nbt.2282 22820318 PMC3467340

[64] Sasagawa Y Nikaido I Hayashi T Danno H Uno KD Imai T et al. Quartz-Seq: a highly reproducible and sensitive single-cell RNA sequencing method, reveals non-genetic gene-expression heterogeneity. *Genome Biol.* (2013) 14:R31. 10.1186/gb-2013-14-4-r31 23594475 PMC4054835

[65] Islam S Kjällquist U Moliner A Zajac P Fan JB Lönnerberg P et al. Highly multiplexed and strand-specific single-cell RNA 5’ end sequencing. *Nat Protoc.* (2012) 7:813–28. 10.1038/nprot.2012.022 22481528

[66] Goetz JJ Trimarchi JM. Transcriptome sequencing of single cells with Smart-Seq. *Nat Biotechnol.* (2012) 30:763–5. 10.1038/nbt.2325 22871714

[67] Zhu YY Machleder EM Chenchik A Li R Siebert PD. Reverse transcriptase template switching: a SMART approach for full-length cDNA library construction. *Biotechniques.* (2001) 30:892–7. 10.2144/01304pf02 11314272

[68] Fu GK Hu J Wang PH Fodor SP. Counting individual DNA molecules by the stochastic attachment of diverse labels. *Proc Natl Acad Sci U S A.* (2011) 108:9026–31. 10.1073/pnas.1017621108 21562209 PMC3107322

[69] Wang J Zhu N Su X Gao Y Yang R. Novel tumor-associated macrophage populations and subpopulations by single cell RNA sequencing. *Front Immunol.* (2023) 14:1264774. 10.3389/fimmu.2023.1264774 38347955 PMC10859433

[70] Liao J Lu X Shao X Zhu L Fan X. Uncovering an organ’s molecular architecture at single-cell resolution by spatially resolved transcriptomics. *Trends Biotechnol.* (2021) 39:43–58. 10.1016/j.tibtech.2020.05.006 32505359

[71] Srivatsan SR Regier MC Barkan E Franks JM Packer JS Grosjean P et al. Embryo-scale, single-cell spatial transcriptomics. *Science.* (2021) 373:111–7. 10.1126/science.abb9536 34210887 PMC9118175

[72] Wu F Fan J He Y Xiong A Yu J Li Y et al. Single-cell profiling of tumor heterogeneity and the microenvironment in advanced non-small cell lung cancer. *Nat Commun.* (2021) 12:2540. 10.1038/s41467-021-22801-0 33953163 PMC8100173

[73] van Galen P Hovestadt V Wadsworth Ii MH Hughes TK Griffin GK Battaglia S et al. Single-cell RNA-seq reveals AML hierarchies relevant to disease progression and immunity. *Cell.* (2019) 176:1265.e–81.e. 10.1016/j.cell.2019.01.031 30827681 PMC6515904

[74] Campbell KA Colacino JA Puttabyatappa M Dou JF Elkin ER Hammoud SS et al. Placental cell type deconvolution reveals that cell proportions drive preeclampsia gene expression differences. *Commun Biol.* (2023) 6:264. 10.1038/s42003-023-04623-6 36914823 PMC10011423

[75] Muñoz-Blat I Pérez-Moraga R Castillo-Marco N Cordero T Ochando A Ortega-Sanchís S et al. Multi-omics-based mapping of decidualization resistance in patients with a history of severe preeclampsia. *Nat Med.* (2025) 31:502–13. 10.1038/s41591-024-03407-7 39775038 PMC11835751

[76] Solt I Cohen SM Admati I Beharier O Dominsky O Yagel S. Placenta at single-cell resolution in early and late preeclampsia: insights and clinical implications. *Am J Obstet Gynecol.* (2025) 232:S176–89. 10.1016/j.ajog.2025.01.041 40253080

[77] Fei H Lu X Shi Z Liu X Yang C Zhu X et al. Deciphering the preeclampsia-specific immune microenvironment and the role of pro-inflammatory macrophages at the maternal-fetal interface. *Elife.* (2025) 13:R100002. 10.7554/elife.100002.3.sa0 PMC11952753 40152904

[78] Huhn O Zhao X Esposito L Moffett A Colucci F Sharkey AM. How do uterine natural killer and innate lymphoid cells contribute to successful pregnancy. *Front Immunol.* (2021) 12:607669. 10.3389/fimmu.2021.607669 34234770 PMC8256162

[79] Whettlock EM Woon EV Cuff AO Browne B Johnson MR Male V. Dynamic changes in uterine NK cell subset frequency and function over the menstrual cycle and pregnancy. *Front Immunol.* (2022) 13:880438. 10.3389/fimmu.2022.880438 35784314 PMC9245422

[80] Xiao S Ding Y Yu L Deng Y Zhou Y Peng M et al. Maternal-fetal interface cell dysfunction in patients with preeclampsia revealed via single-cell RNA sequencing. *Am J Reprod Immunol.* (2025) 94:e70101. 10.1111/aji.70101 40924867

[81] Rong M Yan X Zhang H Zhou C Zhang C. Dysfunction of decidual macrophages is a potential risk factor in the occurrence of preeclampsia. *Front Immunol.* (2021) 12:655655. 10.3389/fimmu.2021.655655 34054819 PMC8152936

[82] Couture C Brien ME Rechtzigel J Ling SY Ledezma-Soto C Bishop GD et al. Predictive biomarkers and initial analysis of maternal immune alterations in postpartum preeclampsia reveal an immune-driven pathology. *Front Immunol.* (2024) 15:1380629. 10.3389/fimmu.2024.1380629 38745664 PMC11091301

[83] Jiang P Zhu X Jiang Y Li H Luo Q. Targeting JUNB to modulate M2 macrophage polarization in preeclampsia. *Biochim Biophys Acta Mol Basis Dis.* (2024) 1870:167194. 10.1016/j.bbadis.2024.167194 38663490

[84] Liu Z Pei J Zhang X Wang C Tang Y Liu H et al. CD74 deficiency reduces trophoblast invasion and proliferation mediated by SIRT1 in preeclampsia. *Reproduction.* (2023) 166:423–35. 10.1530/rep-23-0202 37796743

[85] He C Du Y Chen R Qiu Y Huang J Lin L et al. Excess neprilysin in placental EVs impairs CNP-NPRB-mediated vasodilation to trigger preeclamptic hypertension. *Circ Res.* (2025) 136:1526–41. 10.1161/circresaha.124.325673 40304042

[86] Botha SM Bartho LA Hartmann S Cannon P Nguyen A Nguyen TV et al. Cystatin 6 (CST6) and Legumain (LGMN) are potential mediators in the pathogenesis of preeclampsia. *Sci Rep.* (2025) 15:12945. 10.1038/s41598-025-96823-9 40234537 PMC12000359

[87] Wang H Han X Wu Z Guo M Luo S Fang L et al. G protein-coupled estrogen receptor promotes human extravillous trophoblast invasion via YAP-Snail-mediated CYR61 expression. *Cell Signal.* (2025) 135:112033. 10.1016/j.cellsig.2025.112033 40744331

[88] Liu Y Du L Gu S Liang J Huang M Huang L et al. Identification of the role of DAB2 and CXCL8 in uterine spiral artery remodeling in early-onset preeclampsia. *Cell Mol Life Sci.* (2024) 81:180. 10.1007/s00018-024-05212-4 38613672 PMC11016014

[89] Wilson SL Blair JD Hogg K Langlois S von Dadelszen P Robinson WP. Placental DNA methylation at term reflects maternal serum levels of INHA and FN1, but not PAPPA, early in pregnancy. *BMC Med Genet.* (2015) 16:111. 10.1186/s12881-015-0257-z 26654447 PMC4676901

[90] Buckberry S Bianco-Miotto T Bent SJ Dekker GA Roberts CT. Integrative transcriptome meta-analysis reveals widespread sex-biased gene expression at the human fetal-maternal interface. *Mol Hum Reprod.* (2014) 20:810–9. 10.1093/molehr/gau035 24867328 PMC4106635

[91] Clark SJ Argelaguet R Kapourani CA Stubbs TM Lee HJ Alda-Catalinas C et al. scNMT-seq enables joint profiling of chromatin accessibility DNA methylation and transcription in single cells. *Nat Commun.* (2018) 9:781. 10.1038/s41467-018-03149-4 29472610 PMC5823944

[92] Li J Wang X Lackner AI Narasimhan P Li L Mallajosyula V et al. Regulatory KIR(+)CD8(+) T cells are elevated during human pregnancy. *Sci Transl Med.* (2025) 17:eadm7697. 10.1126/scitranslmed.adm7697 40768597 PMC12696559

[93] Huang Z Zhang P Chen R Sun L Wang J Yan R et al. Targeting A2M-LRP1 reverses uterine spiral artery remodeling disorder and alleviates the progression of preeclampsia. *Cell Commun Signal.* (2025) 23:107. 10.1186/s12964-025-02060-y 39994728 PMC11852582

[94] Huang Y Zhao S Zeng C Shi S Li Z Shen L et al. c-Fos mediates preeclampsia through p-AMPK/detyrosinated tubulin pathway. *Hypertension.* (2025) 82:1959–74. 10.1161/hypertensionaha.124.24416 40859841

[95] Zhong R Guo Y Huang J Yang Y Ren S Gu Y et al. Insights into preeclampsia: a bioinformatics approach to deciphering genetic and immune contributions. *Front Genet.* (2024) 15:1372164. 10.3389/fgene.2024.1372164 39165753 PMC11333266

[96] Ruan F Wang Y Ying X Liu Y Xu J Zhao H et al. Bioinformatics analysis of shared biomarkers and immune pathways of preeclampsia and periodontitis. *BMC Pregnancy Childbirth.* (2025) 25:217. 10.1186/s12884-025-07277-w 40016711 PMC11866586

[97] Bernier E Couture C Borchers A Brien ME Graham CH Girard S. Circulating immune cells from early- and late-onset pre-eclampsia displays distinct profiles with differential impact on endothelial activation. *J Immunol.* (2024) 213:1292–304. 10.4049/jimmunol.2400196 39302114 PMC11491498

[98] Zheng S Feng W Sun Z Xu P Dong S Pan L et al. HSD17B1-mediated trophoblast differentiation lowers estrogen levels in early-onset preeclampsia. *Sci Rep.* (2025) 15:17448. 10.1038/s41598-025-02490-1 40394177 PMC12092795

[99] Kadam L Jain C Kohan-Ghadr HR Krawetz SA Drewlo S Armant DR. Endocervical trophoblast for interrogating the fetal genome and assessing pregnancy health at five weeks. *Eur J Med Genet.* (2019) 62:103690. 10.1016/j.ejmg.2019.103690 31226440 PMC6652202

[100] Xiong Z Guan H Pei S Wang C. Identification of metabolism-related subtypes and feature genes of pre-eclampsia. *Sci Rep.* (2025) 15:4986. 10.1038/s41598-025-89140-8 39930027 PMC11811273

[101] Liu L Li X Yang H Xu F Dong X. Bioinformatic analysis of apoptosis-related genes in preeclampsia using public transcriptomic and single-cell RNA sequencing datasets. *J Inflamm Res.* (2025) 18:4785–812. 10.2147/jir.s507660 40224388 PMC11992479

[102] Tarca AL Romero R Erez O Gudicha DW Than NG Benshalom-Tirosh N et al. Maternal whole blood mRNA signatures identify women at risk of early preeclampsia: a longitudinal study. *J Matern Fetal Neonatal Med.* (2021) 34:3463–74. 10.1080/14767058.2019.1685964 31900005 PMC10544754

[103] Sun L Shi M Wang J Han X Wei J Huang Z et al. Overexpressed trophoblast glycoprotein contributes to preeclampsia development by inducing abnormal trophoblast migration and invasion toward the uterine spiral artery. *Hypertension.* (2024) 81:1524–36. 10.1161/hypertensionaha.124.22923 38716674

[104] Guo F Zhang B Yang H Fu Y Wang Y Huang J et al. Systemic transcriptome comparison between early- and late-onset pre-eclampsia shows distinct pathology and novel biomarkers. *Cell Prolif.* (2021) 54:e12968. 10.1111/cpr.12968 33332660 PMC7848957

[105] Liu Y Fan X Wang R Lu X Dang YL Wang H et al. Single-cell RNA-seq reveals the diversity of trophoblast subtypes and patterns of differentiation in the human placenta. *Cell Res.* (2018) 28:819–32. 10.1038/s41422-018-0066-y 30042384 PMC6082907

[106] Suryawanshi H Morozov P Straus A Sahasrabudhe N Max KEA Garzia A et al. A single-cell survey of the human first-trimester placenta and decidua. *Sci Adv.* (2018) 4:eaau4788. 10.1126/sciadv.aau4788 30402542 PMC6209386

[107] Vento-Tormo R Efremova M Botting RA Turco MY Vento-Tormo M Meyer KB et al. Single-cell reconstruction of the early maternal-fetal interface in humans. *Nature.* (2018) 563:347–53. 10.1038/s41586-018-0698-6 30429548 PMC7612850

[108] Chang X Zheng Y Xu K. Single-cell RNA sequencing: technological progress and biomedical application in cancer research. *Mol Biotechnol.* (2024) 66:1497–519. 10.1007/s12033-023-00777-0 37322261 PMC11217094

[109] Zhang L Li Z Skrzypczynska KM Fang Q Zhang W O’Brien SA et al. Single-cell analyses inform mechanisms of myeloid-targeted therapies in colon cancer. *Cell.* (2020) 181:442.e–59.e. 10.1016/j.cell.2020.03.048 32302573

[110] Derisoud E Jiang H Zhao A Chavatte-Palmer P Deng Q. Revealing the molecular landscape of human placenta: a systematic review and meta-analysis of single-cell RNA sequencing studies. *Hum Reprod Update.* (2024) 30:410–41. 10.1093/humupd/dmae006 38478759 PMC11215163

[111] Garcia-Flores V Romero R Tarca AL Peyvandipour A Xu Y Galaz J et al. Deciphering maternal-fetal cross-talk in the human placenta during parturition using single-cell RNA sequencing. *Sci Transl Med.* (2024) 16:eadh8335. 10.1126/scitranslmed.adh8335 38198568 PMC11238316

[112] Pique-Regi R Romero R Tarca AL Sendler ED Xu Y Garcia-Flores V et al. Single cell transcriptional signatures of the human placenta in term and preterm parturition. *Elife.* (2019) 8:e52004. 10.7554/elife.52004 31829938 PMC6949028

[113] Tsang J Vong J Ji L Poon LCY Jiang P Lui KO et al. Integrative single-cell and cell-free plasma RNA transcriptomics elucidates placental cellular dynamics. *Proc Natl Acad Sci U S A.* (2017) 114:E7786–95. 10.1073/pnas.1710470114 28830992 PMC5604038

[114] Wang Q Li J Wang S Deng Q An Y Xing Y et al. Single-cell transcriptional profiling reveals cellular and molecular divergence in human maternal-fetal interface. *Sci Rep.* (2022) 12:10892. 10.1038/s41598-022-14516-z 35764880 PMC9240006

[115] Arutyunyan A Roberts K Troulé K Wong FCK Sheridan MA Kats I et al. Spatial multiomics map of trophoblast development in early pregnancy. *Nature.* (2023) 616:143–51. 10.1038/s41586-023-05869-0 36991123 PMC10076224

[116] Gao L Mathur V Tam S Zhou X Cheung MF Chan LY et al. Single-cell analysis reveals transcriptomic and epigenomic impacts on the maternal-fetal interface following SARS-CoV-2 infection. *Nat Cell Biol.* (2023) 25:1047–60. 10.1038/s41556-023-01169-x 37400500 PMC10344786

[117] Pique-Regi R Romero R Tarca AL Luca F Xu Y Alazizi A et al. Does the human placenta express the canonical cell entry mediators for SARS-CoV-2. *Elife.* (2020) 9:e58716. 10.7554/elife.58716 32662421 PMC7367681

[118] Wang M Liu Y Sun R Liu F Li J Yan L et al. Single-nucleus multi-omic profiling of human placental syncytiotrophoblasts identifies cellular trajectories during pregnancy. *Nat Genet.* (2024) 56:294–305. 10.1038/s41588-023-01647-w 38267607 PMC10864176

